# Navigating Fibrous Dysplasia of the Middle Turbinate: A Decade-Long Journey and Comprehensive Management Strategies

**DOI:** 10.7759/cureus.51313

**Published:** 2023-12-30

**Authors:** Ghada AlAmmari, Aeshah Almahbub, Fahad Alfawwaz

**Affiliations:** 1 Otolaryngology-Head and Neck Surgery, King Fahad Medical City, Riyadh, SAU

**Keywords:** rhinitis, nasal obstruction surgery, fibersosseous, sinonasal tumor, fibrous dysplasia (fd)

## Abstract

The authors report an unusual case of monostotic fibrous dysplasia involving the middle turbinate. Only four cases of fibrous dysplasia (FD) involving the middle turbinate had previously been documented globally, all of which entailed severe presentation and were treated surgically. We report the first case of asymptomatic monostotic FD of the middle turbinate in the Middle East, followed over a span of 10 years.

Fibro-osseous lesions are rare, benign tumors of the sinonasal and orbital regions. Craniofacial FD most commonly affects the maxilla and mandible, followed by the frontal, parietal, and occipital bones. The prevalence of most patients presenting with monostotic craniofacial FD is between 10% and 29%. It is typically found incidentally, and is most likely to be asymptomatic. The risk of skull base damage and cerebrospinal fluid leaks is increased in this case due to the involvement of the middle turbinate and the expansion of FD into the lateral lamella and the cribriform plate.

Our patient was managed conservatively with regular follow-up due to the minor clinical symptoms despite the extent of the disease. We aim to elucidate the specific challenges associated with middle turbinate involvement and contribute to the growing body of knowledge in this field, ultimately improving the care and quality of life for individuals affected by this condition.

## Introduction

Fibrous dysplasia (FD) has been regarded as a sporadic benign skeletal disease that can affect one bone (monostotic form) or multiple bones (polyostotic form) [1-3]. The monostotic form is more common and accounts for 70% of all cases, affecting the second to third decade of life, whereas polyostotic FD accounts for 30% of all cases, affecting children younger than 10 years of age. The lesions grow with the child and plateau during puberty years [1]. Moreover, there is an equivalent occurrence rate of both monostotic and polyostotic forms in both males and females [1,2]. It is reported that lesions may involve craniofacial bones, ribs, metaphysis, or diaphysis of the proximal femur or tibia, which would give rise to different clinical disorders that might need multidisciplinary collaborative efforts between otolaryngologists, neurosurgeons, and orthopedic specialists to ensure optimal outcomes for affected individuals [1-3]. FD is generally asymptomatic at the onset; the clinical presentation depends on the involved bone [2]. Craniofacial involvement may lead to nasal obstruction, headaches, and facial asymmetry [2,3].

Isolated involvement of the middle turbinate predisposes a unique situation as there is involvement with the skull base [3]. Because of the critical regional anatomy, it raises additional concerns for the management of middle turbinate involvement and increases surgical morbidity [3]. Contrary to the inferior turbinate's documented involvement, which has been highlighted in several studies [4-6], it does not lead to notable difficulties in management.

The authors present a rare case of monostotic fibrous dysplasia (FD) affecting the middle turbinate. Globally, only four prior cases of FD in the middle turbinate have been documented, all of which presented with severe symptoms and were managed through surgery. In this 10-year follow-up, we document the first case of asymptomatic monostotic FD involving the middle turbinate in Saudi Arabia.

## Case presentation

**Figure 1:**
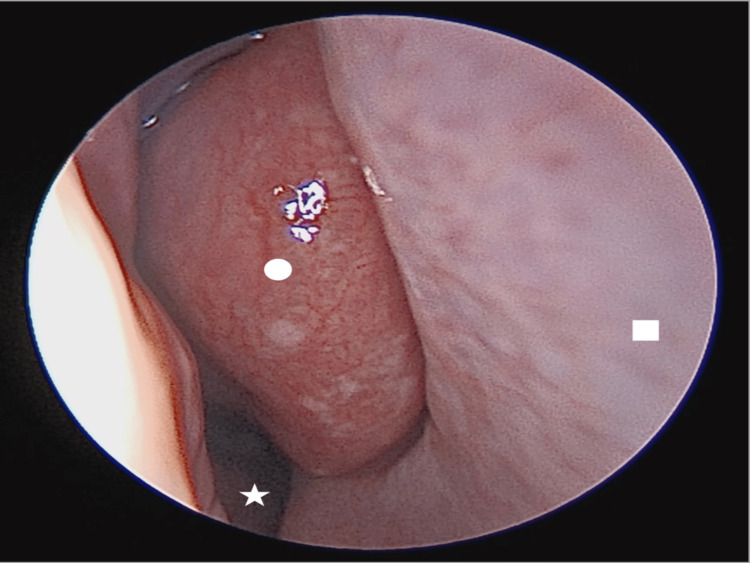
Right nasal cavity anterior endoscopic view showing enlarged middle turbinate (circle), nasal septum (square), inferior turbinate (star).

**Figure 2:**
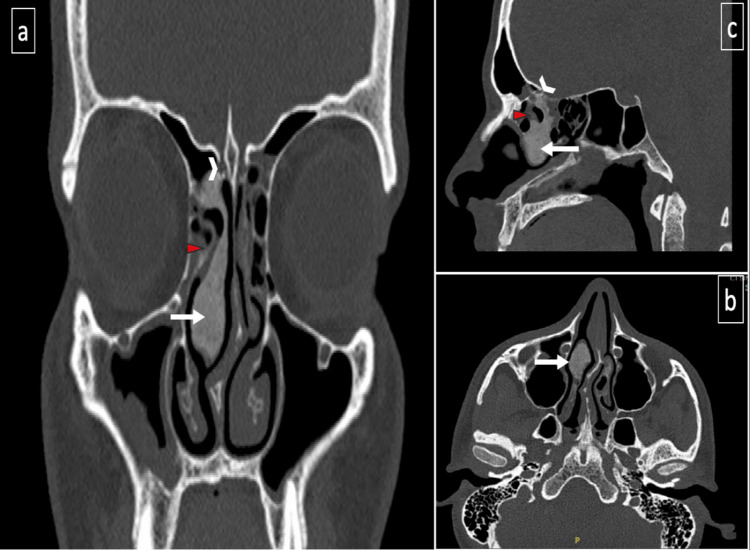
Non-contrast-enhanced computerized tomography of paranasal sinuses performed in 2022 (panels a-c): demonstrate an osseous lesion involving the right middle nasal turbinate (white arrow), extending into the lateral lamella of the right cribriform plate (arrowhead), and obstructing the right anterior ethmoid sinus (red triangle).

A 22-year-old male, medically free, was referred to the ear, nose, and throat (ENT) clinic due to chronic bilateral alternating nasal obstruction and accompanying extra nasal allergic symptoms. He did not report having symptoms of headache, watery rhinorrhea, visual impairment, hyposmia, postnasal drip, or facial pressure. His family history was unremarkable. There was no surgical history before this visit. Additionally, there is no record of any previous trauma. An endoscopic examination using a 0-degree rigid nasal scope revealed a moderately enlarged right middle turbinate, bilateral moderate hypertrophic inferior turbinate, a right anterior nasal septum spur, and thin mucoid discharge (Figure 1). Paranasal sinus computerized tomography (PNS CT) (Figure 2) was performed in late 2022 and showed an osseous lesion involving the right middle turbinate, extending into the lateral lamella of the right cribriform plate and right ethmoid sinus, representing fibrous dysplasia. Magnetic resonance imaging (MRI) could not be performed as the patient had metallic braces. Routine laboratory parameters, including levels of alkaline phosphatase, were normal. Intranasal corticosteroid sprays were prescribed to treat the rhinitis, and the patient reported a satisfactory response to medical treatment for the last 10 years. 

## Discussion

We encountered a case that deviated from the norm found in the literature, as the majority of the reported cases had severe presentations and were managed with a surgical approach. Instead, our case was managed conservatively over the span of 10 years, with a focus on exploring the management options connected to skull base involvement. Fibro-osseous lesions, encompassing fibrous dysplasia and ossifying fibroma, along with their histological variants, represent infrequent, benign tumors found in the sinonasal and orbital regions [4]. They are typically discovered incidentally through radiographic examinations [4]. Fibrous dysplasia (FD) is a skeletal condition that constitutes approximately 7.5% of benign neoplastic bone lesions [1]. There are two clinical subtypes: monostotic and polyostotic [4]. A rarer form presents as a disseminated disease in conjunction with McCune-Albright syndrome, which includes extraskeletal manifestations like precocious puberty, early skeletal maturation, and skin pigmentation alterations [5]. Accurate differential diagnosis is crucial for an effective treatment plan. In cases of ossifying fibroma, complete surgical resection is recommended, as this tumor tends to exhibit more aggressive behavior compared to FD [6].

Craniofacial fibrous dysplasia most commonly affects the maxilla and mandible, with the frontal, parietal, and occipital bones following in prevalence [2,7]. Patients with craniofacial fibrous dysplasia are typically discovered incidentally and are often asymptomatic [7]. The primary clinical symptom of craniofacial fibrous dysplasia is bone discomfort or deformity [7]. However, when the affected bone encroaches on neighboring anatomical structures, it can lead to symptoms such as headaches, vision loss, proptosis, double vision, hearing loss, loss of sense of smell (anosmia), nasal congestion, nosebleeds (epistaxis), excessive tearing (epiphora), and recurrent rhinosinusitis [7]. While the exact incidence of nasal cavity involvement is not well-established, many patients with nasal cavity involvement exhibit symptoms [3].

**Figure 3:**
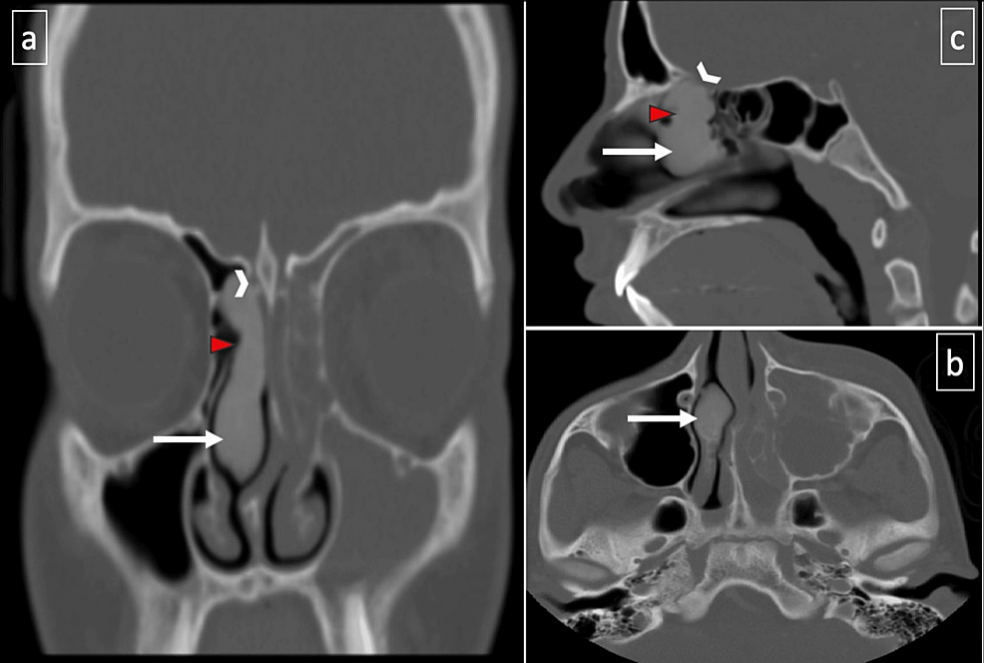
Non-contrast-enhanced computerized tomography of paranasal sinuses, 2013 (panels a-c): demonstrate an osseous lesion involving the right middle nasal turbinate (white arrow), extending into the lateral lamella of the right cribriform plate (arrowhead), and obstructing the right anterior ethmoid sinus (red triangle). On the contralateral side, left anterior ethmoid and maxillary sinusitis can be observed.

Upon reviewing the patient's medical records, it was noted that an emergency visit occurred in 2013 due to acute left sinusitis (contralateral side) and orbital cellulitis. During this episode, the patient was treated with medical treatment and discharged home; a paranasal sinus (PNS) CT scan was performed, revealing opacifications in the left ethmoid, frontal, and maxillary sinuses, as well as a left subperiosteal abscess. Incidentally, the PNS CT image from that presentation also captured fibrous dysplasia in the right middle turbinate, unrelated to the patient's acute condition. In this study, we present the primary image (PNS CT-2013) for comparison (Figure 3).

In contrast to the recent CT scan from 2022, there was no significant progression of the primary disease. This observation supports the notion that even in this anatomical location and its evident involvement in the vital structures of the skull base [7-8], opting for a conservative care strategy and periodic follow-up assessments can be deemed a suitable management approach, considering the tumor's steady progression and the patient's stable clinical condition.

The detection of FD in imaging is variable and highly dependent on the site of involvement as well as the proportion of involved "mineralized bone to fibrous tissue" within the lesion [1,3]. Performing CT scans and MRIs will aid in the identification of distinct subsites' involvement, identifying specific landmarks, as well as detecting the pressure exerted on the surrounding neurovascular tissue. CT images range from generally sclerotic bone alterations to localized osteolytic processes, depending on the degree of mineralization. Due to the substantial fibrous component, FD exhibits hypointensity in T1 and T2 weighted sequences on MRI [3]. Our patient's middle turbinate appearance was easily diagnosed as FD due to the distinct stable ground glass pattern that was evident in three CT PNS images performed in 2013, 2017, and 2022 [7]. When we conducted a comparison among the three images, it became clear that the disease had neither significantly progressed nor extended. This stability in the disease's nature corresponded with the patient's overall uneventful course of illness [1]. 

The risk of skull base damage and cerebrospinal fluid leak is increased in this case due to the involvement of the middle turbinate and the expansion of FD into the lateral lamella and the cribriform plate [8]. Another factor to consider is the possibility of iatrogenic harm to the middle turbinate on the involved right side [8]. According to Freedman et al., the right-sided cerebrospinal fluid leak is exacerbated if the surgeon has right-hand dominance in site-specific procedures [8]. In most cases, monostotic growth stops in adulthood. Some cases of polyostotic form and McCune-Albright syndrome continue to grow even after reaching adulthood. The reason for the continuation of bone growth after adulthood could not be determined [1,2]. This hypothesis could contribute to comprehending the stable and comparatively symptom-free progression of the patient's condition as he aged during this period; he occasionally missed appointments but continued to respond positively to medical management.

The current treatment guidelines lack universal recognition [1]. Fibrous dysplasia is generally asymptomatic at the onset; the manifestation of various symptoms over time depends on the affected bone. It is imperative to investigate and monitor asymptomatic lesions that exhibit no progression, avoiding the development of deformities or functional impairments owing to their infrequent occurrence [1,2]. Although spontaneous sarcomatous transformation of FD has been described, it occurs in less than 1% of cases, with the vast majority of known cases occurring after radiation therapy [4]. In cases where patients experience symptoms such as severe nasal obstruction or facial asymmetry, surgical intervention should be considered [2-4,7]. The goal of surgical treatment is to address or prevent functional issues while also restoring normal facial aesthetics [2,7]. Some reports indicate that early surgical intervention during the active growth phase could unexpectedly stimulate additional growth potential [3,7]. Given the total extent of the condition and the relatively mild clinical symptoms, our patient received conservative management along with regular follow-up.

## Conclusions

Managing monostotic fibrous dysplasia (FD) in the middle turbinate presents a distinctive challenge due to the potential risk of skull base damage. Regular follow-up assessments involving radiographic imaging, including CT and MRI, are essential. These assessments will allow us to closely monitor the progression of the disease and make informed treatment decisions. The approach to management, whether surgical or conservative, should be individualized based on the clinical manifestations. We advocate considering periodic follow-up in cases of clinically stable monostotic FD, as its slowly progressive disease is evident radiologically in long-term follow-up, as we have observed in our case.
